# Functional Outcomes After Surgery for Total Colonic, Long-Segment, Versus Rectosigmoid Segment Hirschsprung Disease

**DOI:** 10.1097/MPG.0000000000003355

**Published:** 2021-11-12

**Authors:** Sanne J. Verkuijl, Rob J. Meinds, Alida F.W. van der Steeg, Wim G. van Gemert, Ivo de Blaauw, Marieke J. Witvliet, Cornelius E.J. Sloots, Ernst van Heurn, Karin M. Vermeulen, Monika Trzpis, Paul M.A. Broens

**Affiliations:** ∗ Department of Surgery, Division of Pediatric Surgery; † Department of Surgery, Anorectal Physiology Laboratory, University of Groningen, University Medical Center Groningen, Groningen; ‡ Department of Gastroenterology and Hepatology, Medisch Spectrum Twente, Enschede; § Department of Pediatric Surgery, Princess Maxima Center for Pediatric Oncology, Utrecht; || Department of Pediatric Surgery, University Medical Centre Maastricht, University of Maastricht, Maastricht; ¶ Department of Surgery, Division of Pediatric Surgery, Radboudumc–Amalia Children's Hospital, Nijmegen; # Department of Pediatric Surgery, Wilhelmina Children's Hospital, University Medical Centre Utrecht, Utrecht; ∗∗ Department of Pediatric Surgery, Erasmus Medical Centre, Sophia Children's Hospital, Rotterdam; †† Department of Pediatric Surgery, Emma Children's Hospital, Academic Medical Centre and VU University Medical Centre, Amsterdam; ‡‡ Department of Epidemiology, University of Groningen, University Medical Center Groningen, Groningen, The Netherlands.

**Keywords:** aganglionosis, constipation, incontinence

## Abstract

**Objectives:**

Knowledge on long-term outcomes in patients with Hirschsprung disease is progressing. Nevertheless, differences in outcomes according to aganglionic lengths are unclear. We compared long-term bowel function and generic quality of life in Hirschsprung patients with total colonic or long-segment versus rectosigmoid aganglionosis.

**Methods:**

In this nationwide, cross-sectional study participants with proven Hirschsprung disease received the Defecation and Fecal Continence questionnaire, and the Child Health Questionnaire Child Form-87, or the WHO Quality of Life-100. We excluded deceased patients, patients who were younger than 8 years, lived abroad, had a permanent enterostomy, or were intellectually impaired.

**Results:**

The study population (*n* = 334) was operated for rectosigmoid (83.9%), long-segment (8.7%), or total colonic aganglionosis (7.5%). Fecal incontinence in general was not significantly different between the three groups, but liquid fecal incontinence was significantly associated with total colonic aganglionosis (odds ratio [OR] = 6.00, 95% confidence interval [CI] 2.07–17.38, *P* = 0.001). Regarding constipation, patients with total colonic or long-segment aganglionosis were less likely to suffer from constipation than the rectosigmoid group (OR = 0.21, 95% CI, 0.05–0.91, *P* = 0.038 and OR = 0.11, 95% CI, 0.01–0.83, *P* = 0.032). Quality of life was comparable between the three groups, except for a lower physical score in children with total colonic aganglionosis (*P* = 0.016).

**Conclusions:**

Over time Hirschsprung patients with total colonic or long-segment aganglionosis do not suffer from worse fecal incontinence in general. A difference in stool consistency may underlie the association between liquid fecal incontinence and total colonic aganglionosis and constipation in patients with rectosigmoid aganglionosis. Despite these differences, generic quality of life is comparable on reaching adulthood.

**An infographic is available for this article at**: .

**What Is Known/What Is New**

**What Is Known**

- Hirschsprung disease may be limited to the rectosigmoid, but in 20% of the cases the aganglionic segment extends more proximally.
- The general consensus is that the longer the aganglionic segment, the greater the morbidity, but comparison based on large groups of patients is lacking.

**What Is New**

- This nationwide, cross-sectional study shows that patients with total colonic or long-segment aganglionosis do not suffer from worse fecal incontinence in general, nor is their generic quality of life impaired over time.
- These insights may be useful for personalized counseling and long-term follow-up of all patients with Hirschsprung disease.

Hirschsprung disease is defined by the congenital absence of ganglion cells in the distal part of the colon (1). This condition is commonly referred to as aganglionosis of the colon (1). In approximately 80% of patients with Hirschsprung disease, aganglionosis is limited to the rectosigmoid. In 10% of the cases, however, the aganglionic segment can extend more proximally or, as seen in another 10%, the total length of the colon is affected (1–3).

Hirschsprung patients often require surgery at an early age to remove the aganglionic, dysfunctional part of the colon using different techniques (4). Unfortunately, long-term bowel dysfunction, such as fecal incontinence and/or constipation, is commonly reported in Hirschsprung patients (3,5–12). Defecatory dysfunction might influence the generic quality of life of Hirschsprung patients and has been reported as being either comparable to (10,12–16) or worse than that of healthy controls (11,17).

The general consensus is that the longer the aganglionic segment, the greater the morbidity. The exact differences, however, in long-term functional outcomes between large groups of patients with total colonic, long-segment, or rectosigmoid aganglionosis have not been analyzed. As a consequence, it is difficult for clinicians to properly counsel patients and their parents about the expected functional outcomes over time, given the length of the aganglionic segment.

We hypothesize that the long-term results and generic quality of life of Hirschsprung patients with long-segment or total colonic aganglionosis will be worse compared to patients with rectosigmoid aganglionosis. Thus, we aimed to compare long-term bowel function and generic quality of life in Hirschsprung patients with total colonic or long-segment versus rectosigmoid aganglionosis.

## METHODS

### Study Design

All six pediatric surgical centers in the Netherlands participated in this cross-sectional study performed between 2018 and 2019. The study included patients from the age of 8 years who had undergone surgery for proven Hirschsprung disease between 1957 and 2015. We excluded patients who had either died, lived abroad, had a permanent enterostomy, or who were intellectually impaired. Patients who gave their informed consent received two questionnaires. In case of patients who were younger than 18 years, we asked the parents or caretakers to complete the questionnaires together with their children. Completion of the full questionnaire was mandatory for the digital questionnaire. Clinical data were extracted from the patients’ medical files. Data from this study population were presented in a previous article (18), in accordance with current data principles (19).

We defined long-segment aganglionosis as an aganglionic segment that extends beyond the sigmoid colon (7,20), but that does not cover the total colon. We excluded ten Hirschsprung patients with ultra-short aganglionic segments because reliable logistic regression analysis of their data was not possible seeing their small number. For the same reason, we excluded another two patients: one had undergone Soave pull-through surgery (coloanal anastomosis with intact rectal muscular cuff) and the other had undergone Swenson's pull-through surgery (coloanal anastomosis with full-thickness removal of the rectum) (4).

### Questionnaires

We sent the Pediatric Defecation and Fecal Continence (P-DeFeC) (21,22) questionnaire and the Child Health Questionnaire Child Form 87 (CHQ-CF87) to the younger than 18-year-olds (23). The adult participants completed the Defecation and Fecal Continence (DeFeC) (21) and the WHO Quality of Life 100 (WHOQOL-100) questionnaires (24). These questionnaires have all been validated for use in the Dutch population (21,22,25,26). The DeFeC questionnaire focuses on symptoms of fecal incontinence and constipation, which are part of different scoring systems and criteria. The wording of the questions in the pediatric questionnaire was adjusted so as to be easily understood by children, but the questions remained similar to the adult version of the questionnaire.

### Assessment of Bowel Function

Fecal incontinence was defined in accordance with the Rome IV criteria: recurrent involuntary loss of any type of feces for at least multiple times per month during the last three months (27). Besides, the following subtypes of fecal incontinence were separately classified: soiling (loss of small amounts of feces), urge incontinence (loss of large amounts of feces when unable to reach the toilet in time), solid incontinence (loss of large amounts of feces without feeling urgency), and liquid incontinence (loss of liquid stool). In addition, we used the incontinence score by Vaizey et al and the Wexner score by Jorge and Wexner (28,29). These scoring systems range from 0–24 and 0–20, respectively. Higher scores reflect more severe fecal incontinence.

Constipation was also defined in accordance with the Rome IV criteria. To meet these criteria patients had to have at least two of the following symptoms: >25% straining on defecation, incomplete defecation, anal blockage, hard or lumpy feces, stool frequency of less than three times a week, use of hands when defecating, and defecation rarely occurs without the use of laxatives (30). For the children in this study we used the Rome IV criteria for adults, because we only included children from the age of 8 years and the study design did not allow for physical examination of fecal mass in the rectum. In addition, we used the Constipation Scoring System by Agachan et al and the Five-Item Score for Obstructed Defecation Syndrome by Renzi and colleagues. These are continuous scoring systems ranging from 0–30 and 0–20, respectively. In both cases, higher scores reflect less favorable outcomes regarding constipation (31,32). Usual stool consistency was measured using the Bristol Stool Scale.

### Assessment of Quality of Life

The CHQ-CF87 is a 87-item questionnaire covering 10 domains and containing two single-item questions about the general status of a child's health (23). All domains are scored on a scale of 0 to 100. The higher the score, the higher the quality of life. The WHOQOL-100 is a 100-item questionnaire used for adults and covers six domains and overall quality of life (24). The scores regarding separate domains vary from 4 to 20 points. A score of 20 indicates highest quality of life. For both questionnaires reference values for the healthy Dutch population were obtained from the literature (33,34).

### Statistical Analysis

Statistical analyses were performed with IBM SPSS Statistics, Version 23.0 (Armonk, NY, USA: IBM Corp.). Categorical variables were compared using Pearson chi-square test. For continuous variables we used Mann-Whitney and Kruskal-Wallis tests. For the analysis of quality of life we used means and one-way ANOVA tests, because it is normal for a Likert scale to have a skewed distribution. Besides, it facilitated interpretability of the data. To test for confounding effects, we applied univariable and multivariable binary logistic regression, for which interactions were checked. Variables tending towards significance (*P* < 0.10) in the univariable regression analysis, or variables with a confounding effect based on the literature, were used in the multivariable regression model. The level of statistical significance was set at *P* < 0.05.

### Ethical Approval

The study was conducted in accordance with the Medical Ethical Review Board of University Medical Center Groningen (Approval code METc 2013/226).

## RESULTS

### Patient Characteristics

A total of 830 patients had undergone surgery for Hirschsprung disease in the participating centers. We excluded 43 patients who had died, 57 patients who either lived abroad or whose postal address was unknown, 25 patients who had a permanent enterostomy, and 86 patients who were intellectually impaired. Out of 619 patients, 346 (55.9%) completed and returned the questionnaires. For dropout analysis we refer to the previous article about this study population (18). After excluding the 12 patients with either an ultra-short aganglionosis or Soave or Swenson's surgery, we arrived at a study population of 334 patients with a median age of 17 (8–45) years at follow-up. This study population consisted of 280 patients with rectosigmoid aganglionosis (83.9%), 29 patients with long-segment aganglionosis (8.7%), and 25 patients with total colonic aganglionosis (7.5%, Table 1).

**TABLE 1:** Patient and clinical characteristics according to different lengths of Hirschsprung disease

	Rectosigmoid no. (%)	Long-segment no. (%)	Total-colonic no. (%)	*P* value^†^
Overall	280 (83.8)	29 (8.7)	25 (7.5)	
Male	225 (80.4)	21 (72.4)	18 (72.0)	*0.405*
Age at follow up (y)^‡^	17.0 (8.0–45.0)	21.0 (10.0–30.0)	16.0 (9.0–36.0)	*0.657*
Age at surgery (mo)^‡^	6.1 (0.5–169.4)	5.4 (0.7–26.1)	4.3 (0.3–88.2)	*0.086*
Other congenital comorbidities	25 (8.9)	4 (13.8)	2 (8.0)	*0.673*
Preoperative enterocolitis	34 (12.1)	4 (13.8)	6 (24.0)	*0.236*
Preoperative enterostomy	118 (42.1)	24 (82.8)	22 (88.0)	*< 0.001* ^∗∗^
Type of reconstruction				*0.416*
Duhamel	175 (62.5)	19 (65.5)	16 (64.0)	
Rehbein	59 (21.1)	5 (17.2)	8 (32.0)	
Transanal endorectal pull-through	46 (16.4)	5 (17.2)	1 (4.0)	
Surgical approach				*0.352*
Laparotomy	196 (70.8)	22 (75.9)	22 (88.0)	
Laparoscopy	35 (12.6)	2 (6.9)	2 (8.0)	
(Combined) transanal	46 (16.0)	5 (17.2)	1 (4.0)	
Postoperative complications	29 (10.4)	4 (13.8)	1 (4.0)	*0.480*
Postoperative enterocolitis	37 (13.2)	3 (10.3)	8 (32.0)	*0.030* ^∗^
Redo pull-through	18 (6.4)	2 (6.9)	2 (8.0)	*0.953*
Anal sphincterotomies	12 (4.3)	1 (3.4)	4 (16.0)	*0.035* ^∗^
Anal dilatation	58 (20.7)	6 (20.7)	8 (32.0)	*0.418*
Permanent enterostomy^§^	23 (3.4)	2 (2.8)	4 (5.6)	*0.597*

† Pairwise comparison of the groups with different aganglionic lengths showed no additional significant differences.

‡ Values are expressed as median ± range.

§ Patients with a permanent enterostomy were excluded, so these prevalences are calculated with the data of all 830 eligible patients.

∗ Statistical significance of *P* < 0.05.

∗∗ Statistical significance of *P* < 0.005.

### Fecal Incontinence Given the Different Lengths of Aganglionosis

The prevalence of fecal incontinence in general did not show a significant difference between the three lengths of aganglionosis (Table 2). Nor did multivariable logistic regression analysis, while adjusted for redo pull-through, age at follow-up, and type of reconstruction (Table 2); however, regression analyses for soiling, liquid stools, urge, and solid incontinence, only showed a significantly increased association with liquid incontinence when the total colonic group was compared to the rectosigmoid group (odds ratio [OR] 6.00, 95% confidence interval [CI], 2.07–17.38, Table 2). For the scores describing the severity of fecal incontinence, we found no significant differences between the three groups of patients with different lengths of aganglionosis (*P* = 0.847 and *P* = 0.687, respectively (see Figure, Supplemental Digital Content 1A).

**TABLE 2:** Prevalence and likelihood of fecal incontinence and constipation

	Prevalence		Univariable logistic regression		Multivariable logistic regression	
	No. (%)	*P* value	Odds ratio (95% CI)	*P* value	Odds ratio (95% CI)	*P* value
Overall fecal incontinence^†^						
Type of Hirschsprung disease						
Rectosigmoid	73 (26.1)	*0.325*	Reference		Reference	
Long-segment	8 (27.6)		1.08 (0.46–2.55)	*0.860*	1.27 (0.52–3.13)	*0.604*
Total-colonic	10 (40.0)		1.89 (0.81–4.39)	*0.139*	1.97 (0.81–4.81)	*0.135*
Soiling^†^						
Type of Hirschsprung disease						
Rectosigmoid	70 (25.0)	*0.725*	Reference		Reference	
Long-segment	8 (27.6)		1.14 (0.49–2.70)	*0.760*	1.30 (0.53–3.19)	*0.566*
Total-colonic	8 (32.0)		1.41 (0.58–3.41)	*0.444*	1.48 (0.59–3.71)	*0.408*
Liquid incontinence^‡^						
Type of Hirschsprung disease						
Rectosigmoid	14 (5.0)	*<0.001* ^∗∗^	Reference			
Long-segment	0 (0.0)		–	*–*		
Total-colonic	6 (24.0)		6.00 (2.07–17.38)	*0.001* ^∗∗^		
Urge incontinence^‡^						
Type of Hirschsprung disease						
Rectosigmoid	9 (3.2)	*0.410*	–	–		
Long-segment	0 (0.0)		–	–		
Total-colonic	0 (0.0)					
Solid incontinence^‡^						
Type of Hirschsprung disease						
Rectosigmoid	13 (4.6)	*0.493*	Reference			
Long-segment	0 (0.0)		–	*–*		
Total-colonic	1 (4.0)		0.86 (0.11–6.83)	*0.883*		
Constipation^§^						
Type of Hirschsprung disease						
Rectosigmoid	67 (23.9)	*0.010* ^∗^	Reference		Reference	
Long-segment	2 (6.9)		0.24 (0.06–1.02)	*0.053*	0.21 (0.05–0.91)	*0.038* ^∗^
Total-colonic	1 (4.0)		0.13 (0.02–1.00)	*0.050* ^∗^	0.11 (0.01–0.83)	*0.032* ^∗^

CI = confidence interval.

† Multivariable analysis was adjusted for redo pull-through, age at follow-up, and type of reconstruction.

‡ Multivariable analysis was not performed because of the limited amount of patients in each group.

§ Multivariable analysis was adjusted for sex, age at follow-up, and type of reconstruction.

∗ Statistical significance of *P* < 0.05.

∗∗ Statistical significance of *P* < 0.005.

### Constipation Given the Different Lengths of Aganglionosis

The prevalence of constipation was significantly higher in the rectosigmoid group compared to the long-segment and total colonic groups (23.9% versus 6.9% and 4.0%, respectively, *P* = 0.010). Also, univariable logistic regression showed that total colonic aganglionosis was significantly associated with less constipation compared to rectosigmoid aganglionosis (*P* = 0.050, Table 2). Subsequently, this result was tested in a multivariable regression analysis in which we adjusted for sex, age at follow-up, and type of reconstruction. This multivariable model revealed that patients with long-segment and total colonic aganglionosis were five and ten times less likely to suffer from constipation in comparison to the rectosigmoid group (OR 0.21, 95% CI, 0.05–0.91 and OR 0.11, 95% CI, 0.01–0.83, respectively, Table 2). Female sex was significantly associated with constipation in the multivariable model. With regard to severity of constipation, the medians of both the Agachan and Renzi scores, which varied between 4.0 and 5.0 points in all three patient groups, were comparable (*P* = 0.425 and *P* = 0.651, respectively, see Figure, Supplemental Digital Content 1B).

### Bowel Symptoms and Bowel Management Given the Different Lengths of Aganglionosis

**FIGURE 1:**
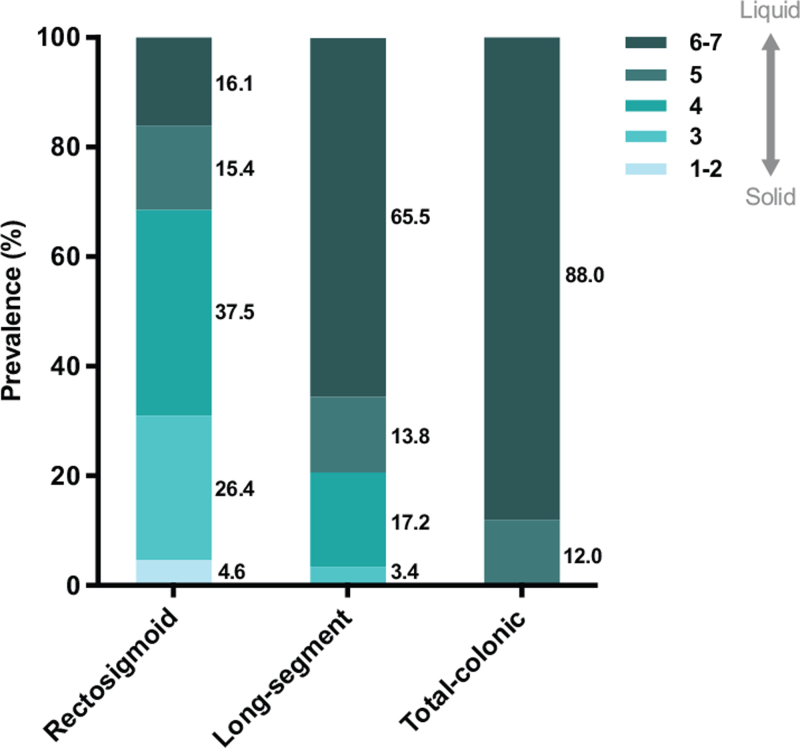
Stool consistency given the lengths of aganglionosis in Hirschsprung disease.

Additionally, we analyzed the separate bowel symptoms (see Figure, Supplemental Digital Content 2). The prevalence of liquid incontinence was significantly higher in the total-colonic group compared to both the rectosigmoid and long-segment groups (24.0% vs 5.0%, *P* < 0.001 and 0.0%, *P* = 0.005, respectively). The prevalence of soiling, solid, and urge fecal incontinence were comparable. We found no difference in the prevalence of any constipation-associated symptoms between patients with the three different lengths of Hirschsprung disease. Furthermore, none of the investigated defecation treatments (colonic irrigations, antidiarrheals, enemas, or laxatives) showed a significant difference between the different lengths of aganglionosis. The use of laxatives did almost reach a statistically significant difference between the rectosigmoid and the total colonic groups (19.3% vs 4.0%, *P* = 0.057, see Figure, Supplemental Digital Content 2). The stool consistency between the three groups of different aganglionic lengths was different: the prevalence of liquid to mushy stool was 88% in the total colonic group versus 65.5% in the long-segment group and 16.1% in the rectosigmoid group (Fig. 1).

### Quality of Life in Children and Adults Given the Different Lengths of Aganglionosis

**FIGURE 2:**
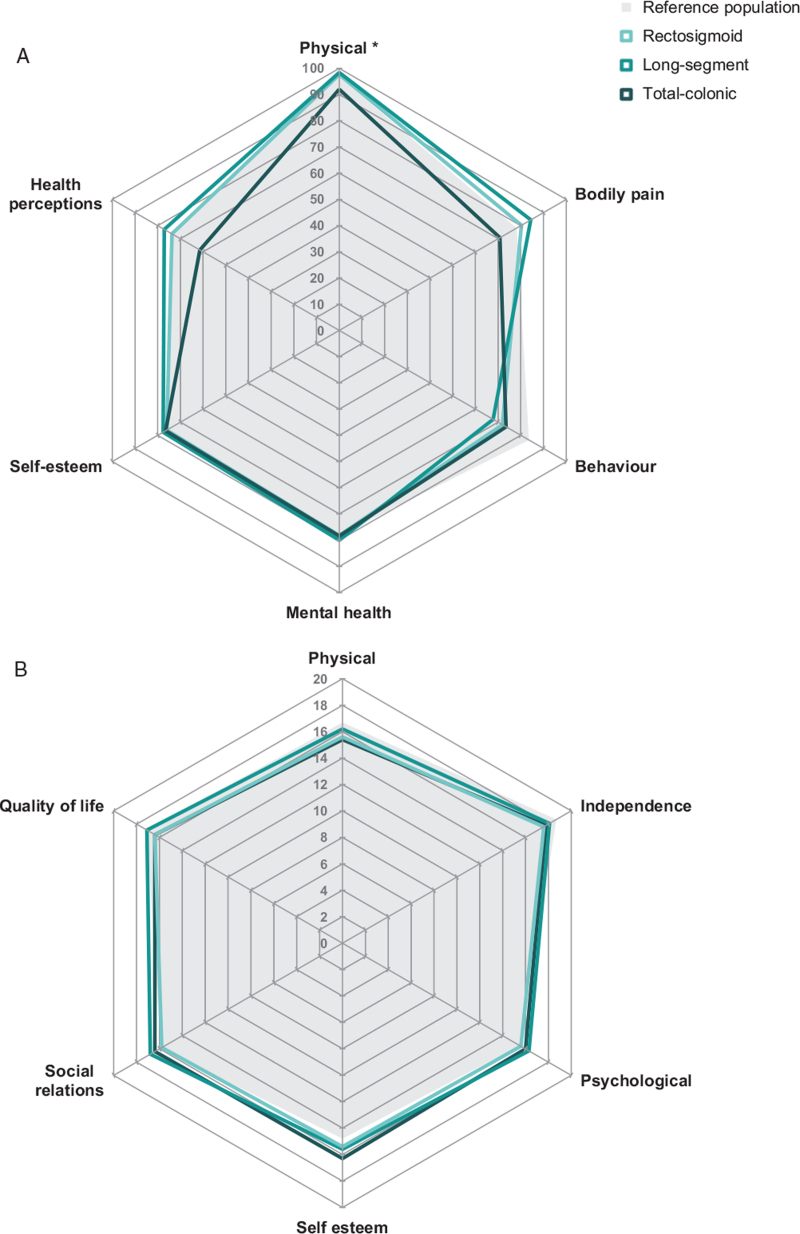
Quality of life scores for children (A) and adults (B) given the lengths of aganglionosis in Hirschsprung's disease. Values were reported as means. CHQ-CF = Child Health Questionnaire Child Form; WHOQOL = WHO Quality of Life.

Quality of life was assessed in 169 patients of 18 years and younger. The physical domain was the only domain that showed a significant difference between the three groups (*P* = 0.046). We found comparable mean scores for the other quality of life domains in the CHQ-CF87 (Fig. 2A). Additionally, comparable reference values for the healthy Dutch population are shown in this figure.

In the quality of life analysis of the 165 adult patients included in our study we considered the mean scores of the WHOQOL-100. Figure 2B illustrates that there were no significant differences between these scores in the three groups with different lengths of aganglionosis and the reference values for the healthy Dutch population.

## DISCUSSION

The present study shows that fecal incontinence in general and the subtype solid fecal incontinence were comparable between patients with total colonic, long-segment, or rectosigmoid aganglionosis. Previously, other investigators reported a prevalence of long-term fecal incontinence of approximately 40% in patients with total colonic aganglionosis, which corresponds with the prevalence found in this study for this group (8,35,36). The range of fecal incontinence in rectosigmoid patients is reported to be between 8% to 71% (2,37). Owing to the many different tools that are used to assess fecal incontinence, this range is broad and it is difficult to compare studies (2,12,38). To enable comparison with other studies we used different continence scoring systems, which all showed that the severity of fecal incontinence between the three groups of aganglionic lengths was comparable.

In contrast to fecal incontinence in general and the subtype solid fecal incontinence, the likelihood for liquid fecal incontinence was six times higher in patients with total colonic aganglionosis compared to patients with rectosigmoid aganglionosis. This may be explained by the more liquid consistency of stool after surgical removal of the total colon, which was also supported by our results. Remarkably, three quarters of the patients with total colonic aganglionosis were continent for liquid stools in the long term, despite the more liquid consistency of their stools.

In all our multivariable regression models of fecal incontinence we corrected for the theoretical confounding effects of age, redo pull-through, and surgical procedure. There is no clarity about the effect of age on fecal incontinence in Hirschsprung patients: improvement (13,15,18,39,40), no effect (5,8–10,12), or even deterioration (3,6), have all been reported. Theoretically, the true prevalence of fecal incontinence could have been underestimated by the use of antidiarrheals or colonic irrigations. There were, however, no significant differences between the use of these types of defecation treatments between the three groups. The endpoint of very severe fecal incontinence may sometimes be a permanent enterostomy. Although patients with a permanent enterostomy were excluded, the prevalence of a permanent enterostomy was comparable between patients with aganglionosis of the rectosigmoid, long-segment, or total colon.

Furthermore, we determined the presence of constipation. Hirschsprung patients with aganglionosis of the rectosigmoid show constipation rates between 1% and 50% (2,37). Our study showed an overall prevalence of constipation of 21.1%. As could be expected from clinical experience, even though we corrected for age, sex, and surgical procedure, patients with rectosigmoid aganglionosis were found to be five and ten times more likely to suffer from constipation than either the long-segment or total colonic groups. Previously, constipation was found to either diminish (5,39,41) or persist (5–7) with age in Hirschsprung disease. It is widely recognized that sex influences constipation (30). Indeed, in our population, sex also had a significant influence on constipation, whereas age did not.

The association of rectosigmoid aganglionosis with constipation could result from the fact that in patients with long-segment or total colonic aganglionosis the colon is removed entirely, while substantial amounts of water are reabsorbed from the stool in this part of the gastrointestinal tract. These patients may therefore have more liquid stool, rendering them less prone to constipation. The use of laxatives was highest in the rectosigmoid group, which is in line with the higher association with constipation in these patients. The fact that the use of laxatives is lower in the long-segment and total colonic groups may explain why the Agachan and Renzi constipation scores are comparable with those of the rectosigmoid group: the use of laxatives weighs more heavily in the Rome IV criteria than in the other two constipation scores (30–32).

Lastly, especially fecal incontinence has previously shown to decrease quality of life (42). Therefore, a decrease in generic quality of life in patients with Hirschsprung's disease was attributed mainly to poor fecal control (10,13,15,16). It was suggested that patients with long-segment and total colonic aganglionosis have lower generic quality of life scores compared to patients with rectosigmoid aganglionosis (43). Other investigators contested this suggestion (12,13,16,44). In the present study, the generic quality of life of children was comparable between the three groups with different lengths of aganglionosis, except for a lower physical function score in the pediatric patients with total colonic aganglionosis. Other researchers also found a lower score in the physical domain in children or adolescents with Hirschsprung disease in comparison to healthy controls (8,14,16). In the adult patients we found no difference between the different domains of the WHOQOL-100, including the physical domain. This might indicate improved recognition of physical symptoms and better adaptation and coping during ageing, which corresponds with previous findings that generic quality of life scores tend to increase with age (8,17,44).

In the present study, the small groups of patients with either long-segment or total colonic aganglionosis was a limitation. Nevertheless, the incidence in this nationwide study cohort resembled the previously reported incidences of the different lengths of aganglionosis (2,3,43,45). This study had an extended follow-up and the groups were large enough to allow acceptable analyses. This is in contrast to previous studies regarding the differences in functional outcomes between patients with rectosigmoid, long-segment, or total colonic aganglionosis, which mainly relied on small sample sizes (7,12,43,46) or only assessed total colonic aganglionosis patients (3,8,35,36,45). Direct comparison of results from studies reporting solely on patients with rectosigmoid, long-segment, or total colonic aganglionosis is problematic because bowel function is difficult to compare on account of the lack of consensus about how it should be assessed (2,15). We used several different scoring systems to simplify comparisons with other studies. Furthermore, this study might have been limited by the use of the Rome IV criteria that were originally developed to diagnose constipation in the absence of physiological or anatomical abnormalities. Nevertheless, we chose to use these criteria because they have been validated and are widely used. The fact that we used the adult version of the Rome IV criteria instead of the pediatric version—even though we adapted the wording of the questions to suit the level of children—may have led us to underestimate the extent of constipation in children. Especially so because the criterion of episodes of fecal incontinence is not included in the adult version. Finally, the clinical data were retrospectively extracted from the patients’ medical files, therefore we may have missed information that had not been recorded at the time.

We conclude that over time Hirschsprung patients with total colonic or long-segment aganglionosis do not suffer from worse fecal incontinence in general, nor is their generic quality of life impaired in comparison to patients with shorter rectosigmoid aganglionosis on reaching adulthood. Apart from the similarities between patients with total colonic, long-segment, or rectosigmoid aganglionosis, stool consistency amongst these patients is different. On the one hand, stool consistency in patients with total colonic aganglionosis is more liquid. As a consequence, several patients in this group suffer from liquid fecal incontinence. On the other hand, over time patients with aganglionosis of the rectosigmoid experience constipation more often. Hopefully, these insights will be useful for personalized counseling and long-term follow-up of all patients with Hirschsprung's disease. Irrespective of whether patients underwent surgery for total colonic, long-segment, or rectosigmoid aganglionosis, addressing the specific bowel function problems they are likely to encounter over time, will help them to cope better with their individual type of Hirschsprung's disease.

## Supplementary Material

Supplemental Digital Content

## Supplementary Material

Supplemental Digital Content

## Supplementary Material

Supplemental Digital Content
