# Supplementary figures and images for: Functional Outcomes After Surgery for Total Colonic, Long-Segment, Versus Rectosigmoid Segment Hirschsprung Disease
Source: J Pediatr Gastroenterol Nutr. 2021 Nov 12;74(3):348–54. doi: 10.1097/MPG.0000000000003355 (PMC8860201; doi:10.1097/MPG.0000000000003355)

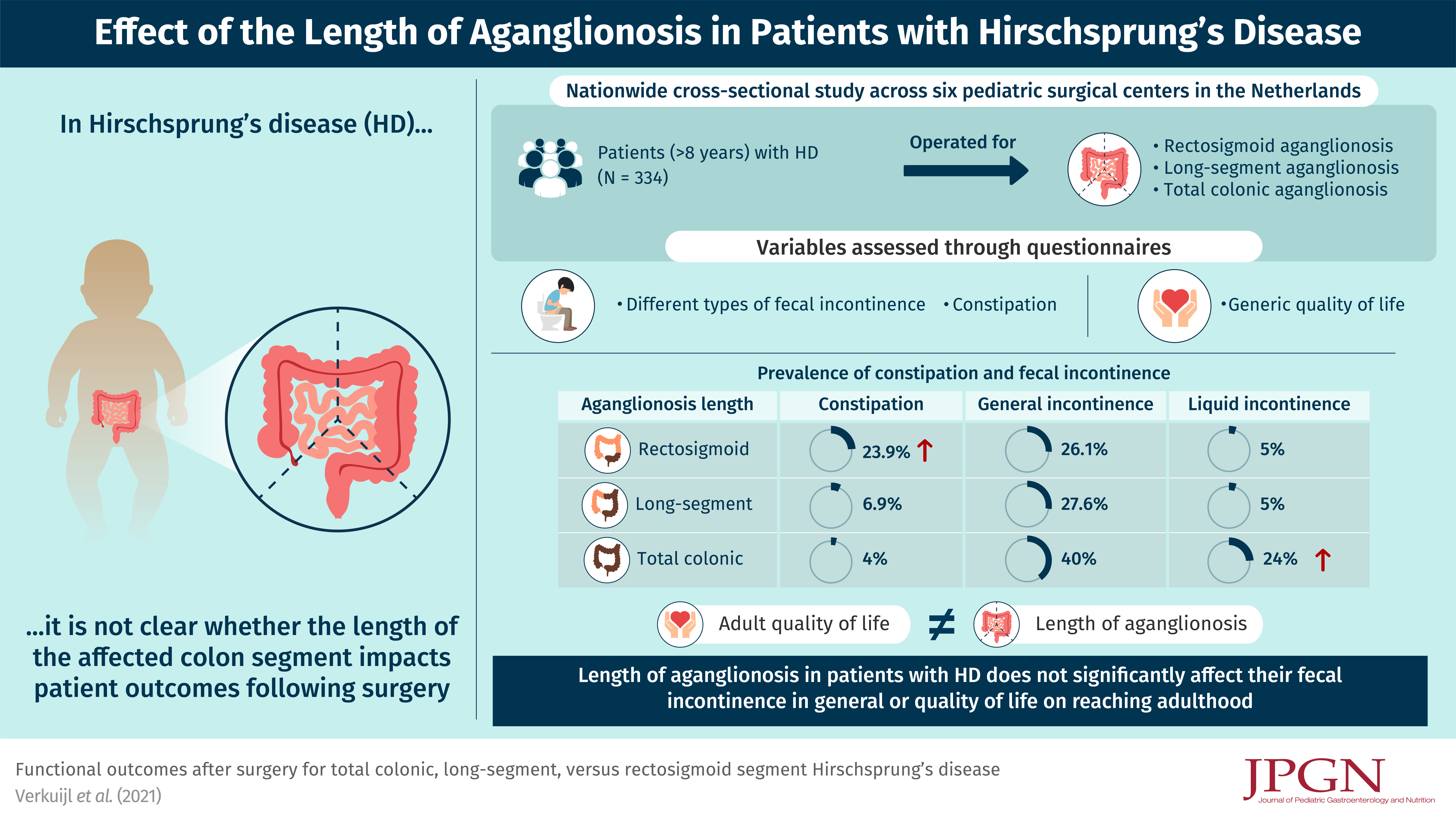

Supplement: Supplemental Digital Content [file jpga-74-348-s001.jpg]

- Rectosigmoid
- Long-segment
- Total-colonic

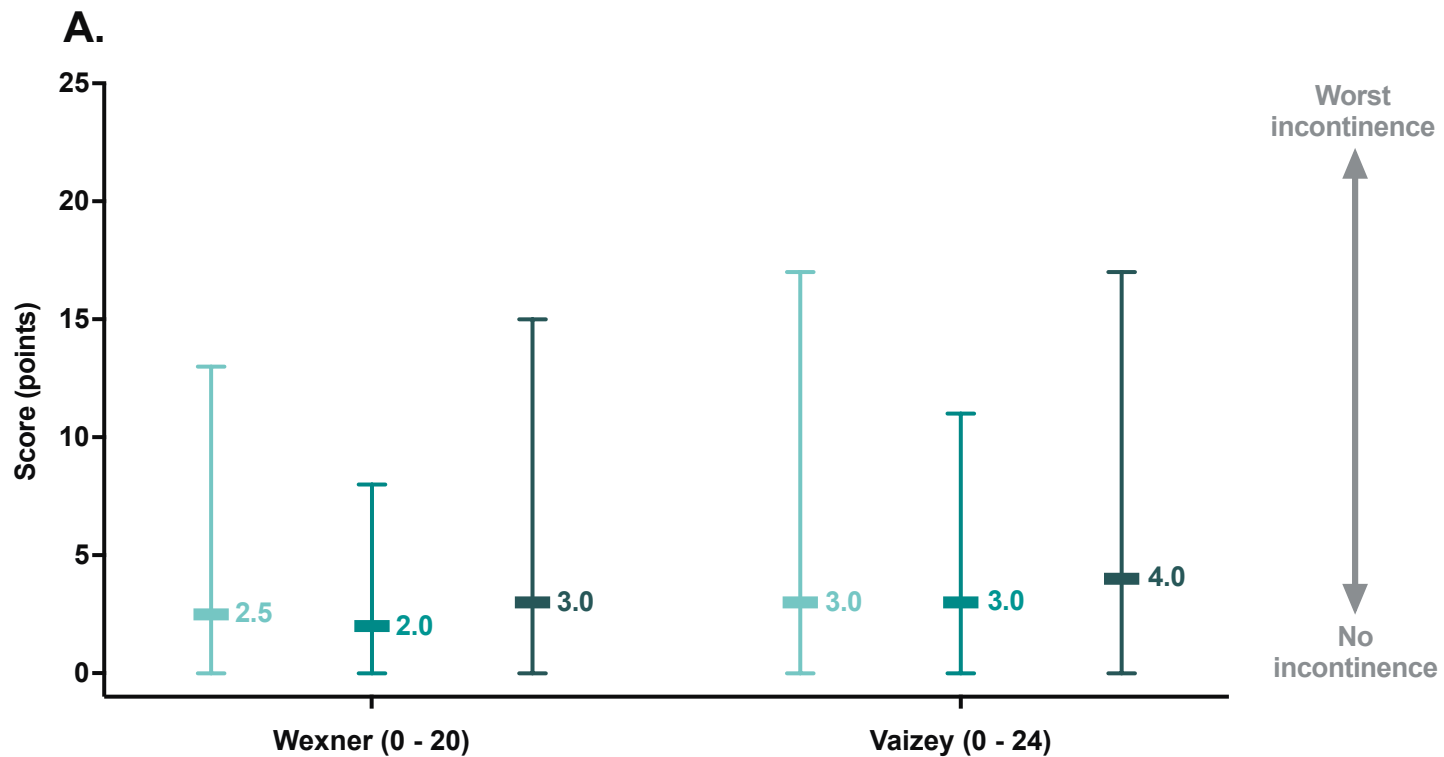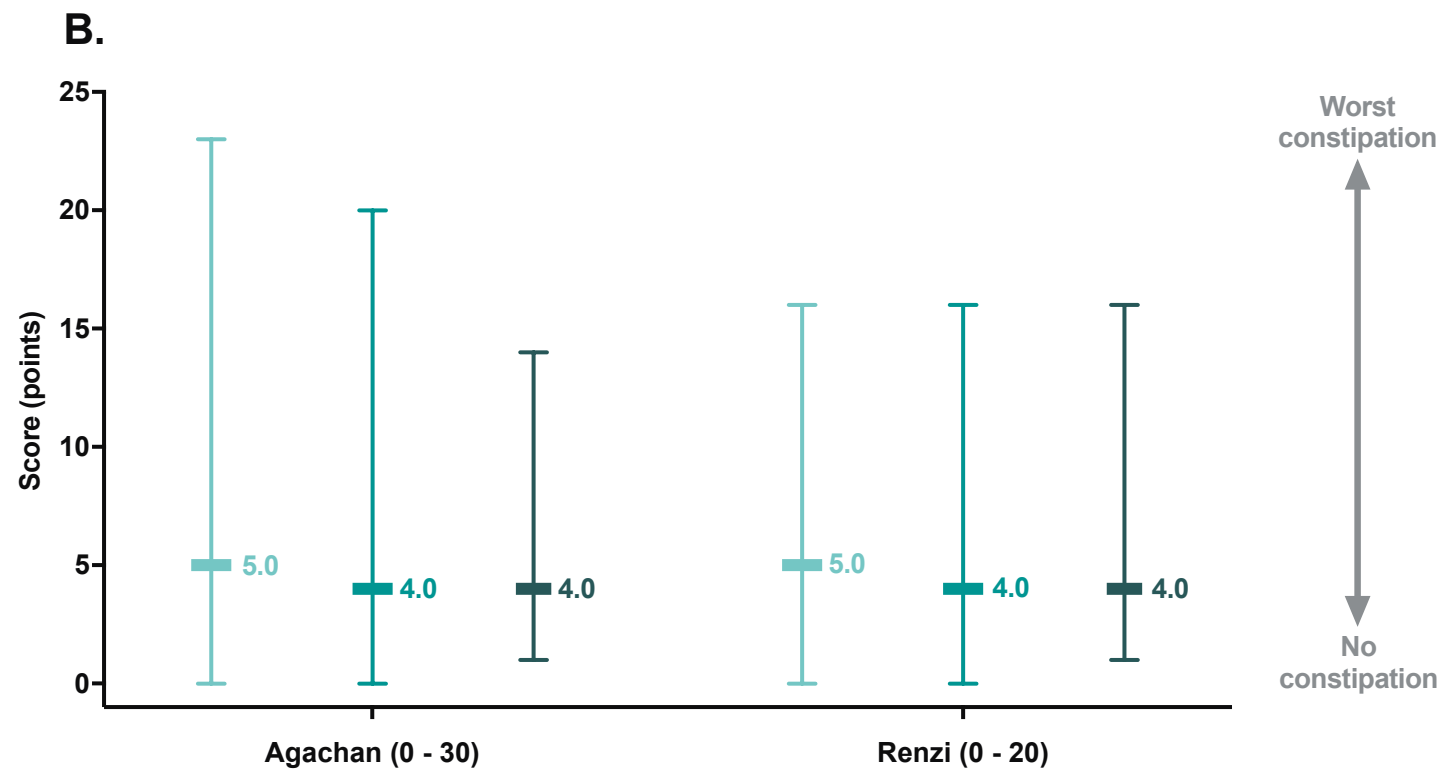

Supplement: Supplemental Digital Content [file jpga-74-348-s002.pdf]

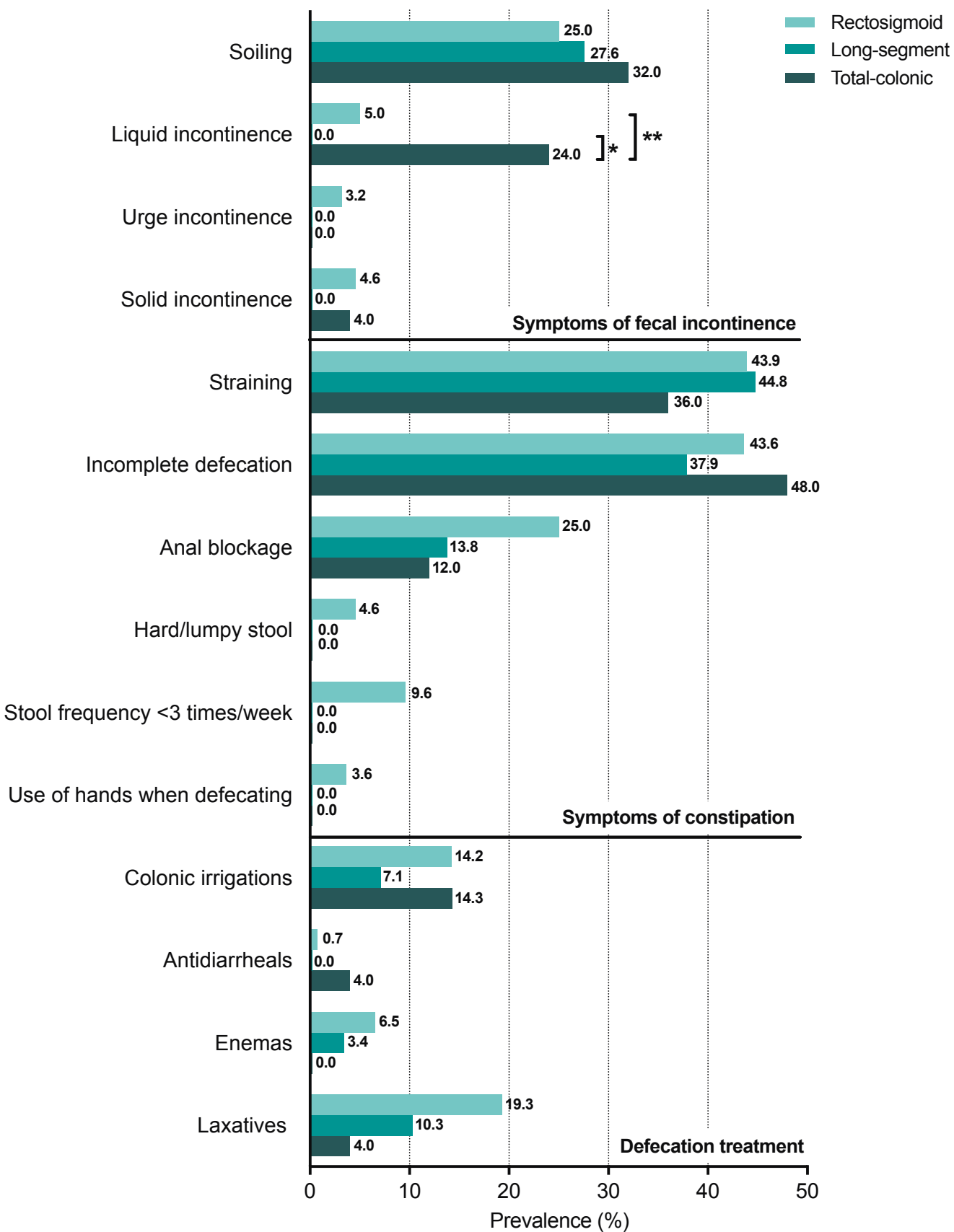

Supplement: Supplemental Digital Content [file jpga-74-348-s003.pdf]
